# 9-point Injection Technique for Lip Augmentation and Lip Corner Lifting Using Sonographic Imaging of the Labial Artery Pathway

**DOI:** 10.1093/asj/sjae086

**Published:** 2024-04-23

**Authors:** Jong Seo Kim

## Abstract

**Background:**

As lip enhancement with fillers has grown in popularity, practitioners have sought to identify injection methods that achieve aesthetically pleasing results while avoiding adverse events such as arterial injury due to intravascular injection.

**Objectives:**

The primary objective of this study was to establish a safe injection technique for creating appealing, proportionate, and aesthetically pleasing lips while elevating the lip corners with filler.

**Methods:**

Before injection, the locations of the superior and inferior labial arteries were established by sonography and a 9-point injection technique (9-PIT) was devised to reliably achieve fashionable lips. Particle hyaluronic acid filler was administered to 50 patients by the 9-PIT and these patients were monitored for 3 months. The extent of lip corner elevation and the angle of lip corners were quantified by 3-dimensional analysis, while changes in the length and curvature along the upper peristomal lines were evaluated after 1 week.

**Results:**

The superior and inferior labial arteries originated from the deep lateral aspect of the lip and gradually traversed toward the midline in the superficial layer. Superficial arterial branches were identified in the submucosal layer near the midline. All patients expressed satisfaction with the lip shaping and corner elevation, without any adverse effects or vascular complications. The angle of lip corners decreased by 8.80%, and lip corners were lifted by 1.02 mm. The upper lip exhibited a more pronounced S-shape, with the upper lip line being elongated by 6.5%. This accentuated S-shape contributed to the appearance of lifted lip corners.

**Conclusions:**

The 9-PIT facilitated safe and aesthetically pleasing lip volumization with corner elevation in a consistent manner, while elucidating vascular pathways. Lip corner elevation was achieved solely using HA filler.

**Level of Evidence: 3:**

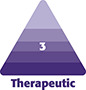

Lip aesthetics has a significant influence on personal self-esteem. Recently, there has been a noticeable increase in the demand for lip augmentation procedures among women. For more than 20 years, the author has been carefully examining the complex pathways of the superior labial artery (SLA) and the inferior labial artery (ILA) by ultrasonography. Additionally, a groundbreaking advance, known as the 9-point injection technique (9-PIT), has been developed to guarantee the safe enhancement of lips.

Numerous studies have highlighted the superior efficacy of particle type hyaluronic acid (p-HA) compared to monophasic HA in lifting lip corners due to its increased firmness and superior elasticity.^1-6^ Conventional surgical interventions aimed at elevating lip corners often lead to noticeable scarring. Although botulinum toxin has been successful in raising lip corners by inhibiting the depressor muscles,^7,8^ this study exclusively investigates the use of HA fillers for this purpose. The focus of the 9-PIT lies in its potential to elevate lip corners while ensuring safety, consistency, aesthetic appeal, and harmonious lip augmentation.

This article outlines a revolutionary procedure to lift and contour the corners of the lips using HA fillers, with a primary focus on safety and aesthetic results.

## METHODS

In 2012, the author pioneered the development of the 9-PIT and has since conducted over 1500 procedures with this protocol. The author diligently captured ultrasound images and 3-dimensional (3D) photographs of all patients before and after the procedures, recognizing the importance of maintaining comprehensive data for both patients and practitioners. In 2017, the author acquired a new ultrasound machine and has consistently observed and recorded ultrasound images before and after filler procedures. A retrospective study was conducted, reviewing 50 cases out of 382 patients treated with the 9-PIT from 2017 to 2022, ensuring sufficient sonography data. This study protocol conformed to the ethical guidelines of the 1975 Declaration of Helsinki as well as to applicable local regulations.

**Figure 1.**
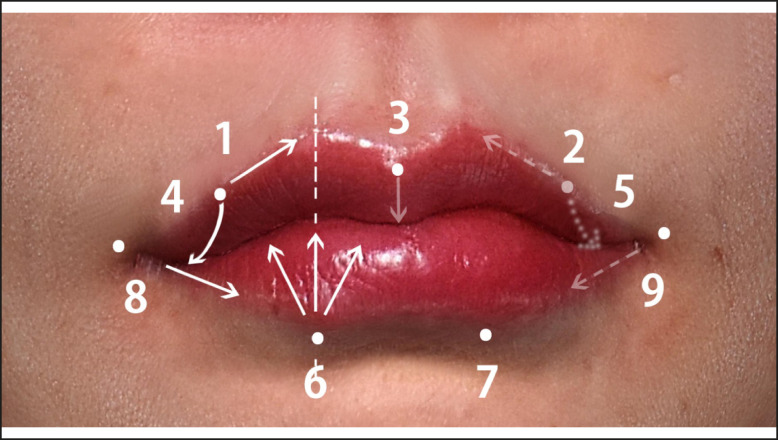
The 9-point injection technique for lip shaping and lip corner lifting. Points 1 and 2 enhance medial upper vermillion border, while Point 3 emphasizes the central tubercle. Points 4 and 5 create new upper lateral tubercles on the lower border of the peristomal zone. Points 6 and 7 enhance both lower tubercles. Points 8 and 9 volumize the lower vermillion border, elongating the lip laterally and providing support to the oral commissures, ultimately lifting the lip corners. The coordination between these points creates a harmonious meeting of the upper and lower lips, resembling gears in occlusion. To achieve optimal lip occlusion, Point 3 should be strategically positioned between Point 6 and 7, Point 4 between Points 6 and 8, Point 5 between Points 7 and 9, Point 6 between Points 3 and 4, and Point 7 between Points 3 and 4. The term "lip occlusion," coined by the author, proves indispensable for both shaping the lips and ensuring their proper functionality.

All patients underwent noninvasive ultrasonography examination before and after the injections to evaluate the pathways of the SLA and ILA. To confirm the depth and location of labial arteries before injection and to ensure a safer injection, the artery depth was also assessed by sonography at the lip corner (0-I)—entry point 1 (I)—apex of Cupid’s bow (II)—midline (III) (Figure 1).

HA filler was administered into the patients’ lips by the 9-PIT in a single session. The 9-PIT injection sites are shown in Figure 1. The lips were examined with a noninvasive 3D scanner 1 week postinjection. Subsequently, all participants were monitored for 3 months to evaluate any changes in lip volume and potential side effects. Using a 29G, 1-inch needle, 20 mg/mL of p-HA filler with 0.3% lidocaine (Yvoire ClassicPlus, LG-Chem, Korea) was injected. The HA filler consisted of small particles ranging from 300 to 400 microns. The standard volume was 1 mL. The HA filler was administered submucosally, avoiding the labial arteries, by the linear thread and fanning technique (Video 1). Molding was only conducted in cases where the injection resulted in asymmetry or necessitated adjustment of the filler's position. Although not all injections could be executed flawlessly, the aim was to achieve optimal results without the need for molding whenever possible. To assess the outcomes following a single injection session, no touch-up or supplementary injections were provided.

### The 9-PIT for Lips

#### Points 1 and 2

**Figure 2.**
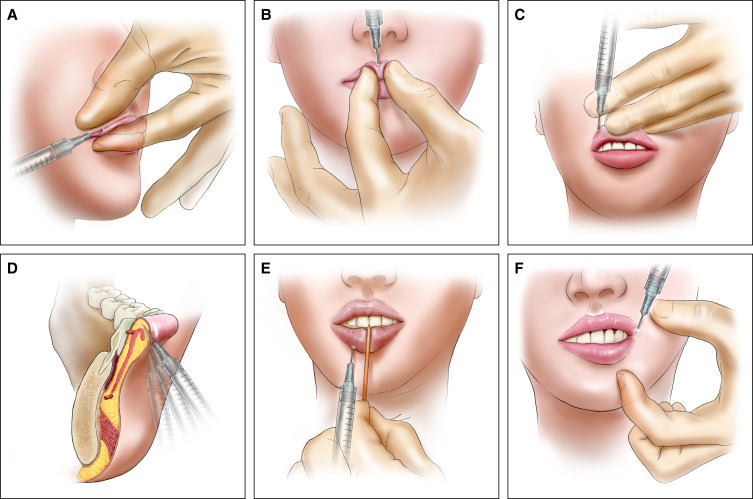
Illustration of the 9-point injection technique. (A) Enhancement of the upper vermilion border by injecting at points 1 and 2 using the pinch technique. (B) Enhancement of the central tubercle by injecting at point 3 using the pinch technique. (C) Creating new upper lateral tubercles by injecting at points 4 and 5. (D) Enhancing the curved submucosal plane on the lower tubercles of the lip (points 6 and 7). (E) Enhancement of the lower tubercle by injecting at points 6 and 7. (F) Supporting and lifting the lip corner by injecting at points 8 and 9.

Entry point 1 was located 1 inch (needle-length) laterally from the right apex of Cupid's bow along the right upper vermilion border. Following the insertion of the needle, the HA filler was submucosally injected to create a distinct ridge using a tight pinch technique, employing linear threads in an anterograde-retrograde injection manner. The amount of injected HA filler was increased near the apex of Cupid's bow. The injection of point 2 was carried out from entry point 2 to the left apex of Cupid's bow along the left upper vermilion (Figures 1, 2; Video 1).

#### Point 3

At point 3, the upper central tubercle was accentuated using the pinch technique to achieve a defined sharp shape. The needle was inserted while injecting HA (anterograde injection) just below the vermilion border and passed just under the mucosa in a downward direction until the needle encountered wet mucosa in the peristoma. Subsequently, a retrograde injection was administered (Figure 2B).

If the filler is placed too deeply, it can lead to a blunt and wide central tubercle. Point 3 should be positioned between points 6 and 7 for “lip occlusion.” For lip occlusion, the corresponding area of the lower lip is not injected. Similarly, when injecting into the upper lip, the vertical imaginary line of the lower lip is not injected. Following the same principle, when injecting into the lower lip, avoid injecting into the corresponding area of the upper lip along the vertical imaginary line. This is the author's rule, implemented to ensure lip occlusion during the 9-PIT procedure.

“Lip occlusion” is a fundamental concept introduced by the author in their innovative approach to lip filler procedures. In many papers, both the upper and lower lips were injected along the same vertical line. However, this practice disrupts “lip occlusion,” resulting in ill-fitting lips and potential water leakage (Supplemental Figure 1).

#### Points 4 and 5

Entry point 1 can also serve as entry point 4. The needle was inserted near entry point 1 and passed to the peristoma caudally and then laterally by a few millimeters. Subsequently, HA filler was injected retrogradely near the wet-dry junction.

By injecting points 4 and 5, new upper lateral tubercles were formed near the peristomal border. Lowering the peristomal border through injection at points 4 and 5 resulted in the creation of an exaggerated S-shaped line, giving the illusion of higher lip corners (oral commissure).^9^ Additionally, a new left upper lateral tubercle was created through injections at point 5 (Figure 2C).

#### Points 6 and 7

Entry point 6 was positioned 1 mm below the vermilion border on the skin, aligning with the vertical imaginary line of the right apex of Cupid's bow. The right lower tubercle was volumized by the fanning technique (between 3 and 5 anterograde linear thread injections [Ar-LTIs] parallel under the mucosa). Due to the curved submucosal plane of the lower tubercle, the needle was maneuvered in a curved fashion. To prevent the spread or migration of the HA filler to the midline and the fusion of the 2 lower tubercles, and to mitigate potential vascular side effects from superficial arteries in the midline, a sterilized toothpick was securely placed on the midline (Figure 2D, E).

#### Points 8 and 9

Entry point 8 was positioned 1 mm lateral to the oral commissure on the skin. Using a 29G, 1-inch needle inserted along the vermilion border, the HA filler was administered by a single retrograde linear thread injection (Re-LTI) to enhance the lower lateral vermilion border. Re-LTI was performed over a 1-inch distance. Volumizing this area has the potential to elongate the lip laterally, providing support and lifting the oral commissures (Figure 2F).

The HA filler was administered by Ar-LTIs in points 1, 2, 3, 6, and 7, excluding points 4, 5, 8, and 9 where Re-LTI was applied. Minimal molding was employed postinjection. Instead of molding, precise injections of the HA were targeted to specific areas, preserving the distinct edges of the vermilion borders. Throughout the 3-month follow-up period, potential adverse effects and the longevity of the HA filler were evaluated by 3D scanner analysis. Additionally, postinjection, ultrasound examination was conducted to assess blood flow obstruction, changes in lip thickness, and the location of the HA filler.

### 3D Image Analysis

3D scanned images were evaluated before and 1 week after the injections utilizing a LifeViz Mini 3D camera (Quantificare, Sophia Antipolis, France). This innovative imaging technology captures noninvasive, in vivo 3D recordings.^10^

### Shape and Extent of Lip Corner Lift

The shape and extent of the lip corner lift were evaluated by the following 4 methods.

#### Angle of Lip Corner

**Figure 3.**
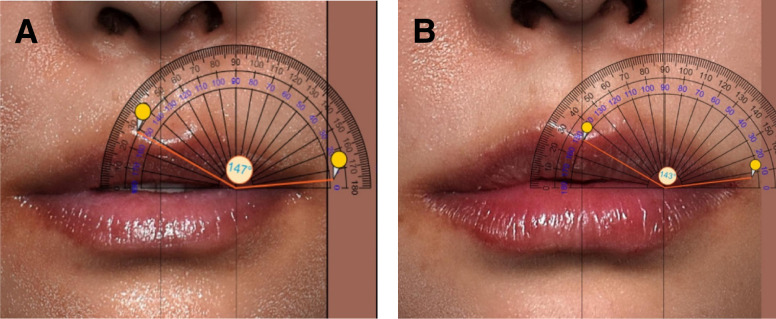
Measuring the angle of the lip corners in a 42-year-old female. The angle of the lip corner is measured between the center of the Cupid's bow, the lowest point in the upper lateral tubercle, and the oral commissure. Initially, the lowest point created in the new upper lateral tubercle after the injection is marked in the 3D image as a vertex (angular) point. This is matched with the corresponding apex at an equidistant point (the same point on the vertical line) in the before image. (A) The angle of lip corner was 147° before the procedure. (B) The angle of lip corner became acute at 143° 1 week after the procedure.

The angle of the lip corner was measured with a digital protractor, with points 4 and 5 serving as a center, between the center of Cupid's bow and the oral commissure^11^ (Figure 3).

#### Extent of Lift of Oral Commissure Using a Computer Program

**Figure 4.**
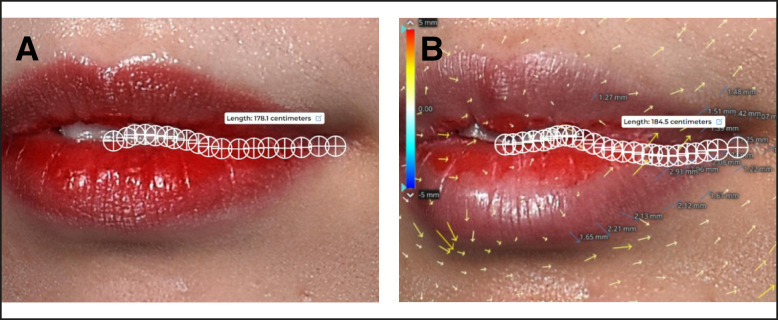
Measuring the extent of lift of the oral commissure and length of the peristomal borderline on the upper lip in a 24-year-old female. The actual extent of lift of the lip corner was measured through 3D scanner analysis. The curved length of the peristomal borderline on the upper lip, measured from the central tubercle to the oral commissure, increased from 178.1 (A) to 184.5 cm 1 week after the procedure (B). The lip shape exhibited an exaggerated S-shape, contributing to the elongation of the peristomal borderline. Furthermore, the 3D analysis revealed an elevation of 1.25 mm in the left oral commissure.

The extent of the lift was measured around the oral commissure by 3D scanner computer analysis. The degree of lift around lip corners was quantified from the 3D images^10^ (Figure 4).

#### Length and Shape Along the Peristomal Border on the Upper Lips

The length of the peristomal border (upper lip wet-dry junction) was measured from the central tubercle to the oral commissure in the frontal view using an online image-measurement program. Additionally, the shape and curvature along the peristomal border on the upper lip were also assessed (Figure 4).

#### Global Aesthetic Improvement Scale (GAIS)

The Global Aesthetic Improvement Scale (GAIS) was used to assess lip shape and corner elevation at 1 week, 1 month, and 3 months posttreatment. Patient satisfaction was rated on a 5-point scale: very much improved (+3), much improved (+2), improved (+1), no change (0), and worse (−1).

## RESULTS

The mean age of participants was 35.7 years (range, 18-65 years), and all participants were female. After receiving p-HA filler injections by the 9-PIT for 3 months, all patients expressed high satisfaction with both the lip shape and lip corner lifting. Notably, no side effects such as redness, delayed swelling, itching, or nodule formation were reported after the injection of p-HA.

### Sonographic Results

**Figure 5.**
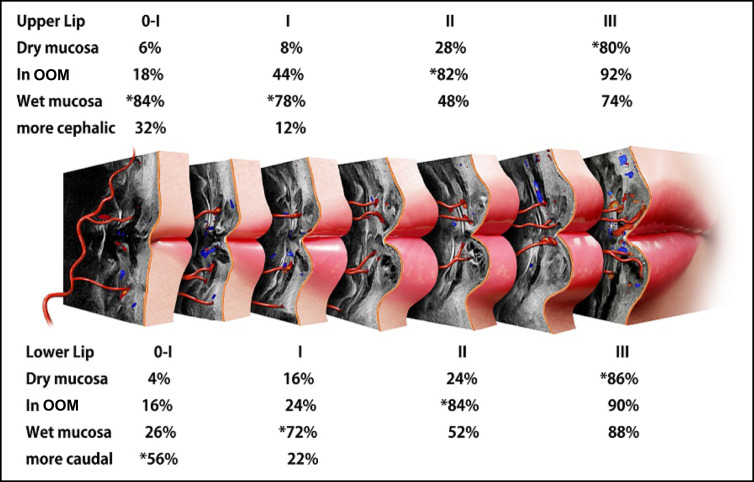
Pathway and depth of the superior and inferior labial arteries. OOM, orbicularis oris muscle. The lower facial artery (FA) bifurcates into ILA behind (laterally) depressor anguli oris (DAO), positioned lateral to and below the lip corner. Subsequently, the ILA travels medially (slightly upward and forward) beneath the DAO and above depressor labii inferioris (DLI). In proximity to the lip corner, the ILA courses under the orbicularis oris muscle (OOM) along wet mucosa. It then pieces pars palpebralis of the OOM in a tortuous course. Continuing, the ILA runs under pars marginalis of the OOM, and over the pars peripheralis of the OOM along the vermillion border of the lower lip. The upper FA branches into the superior laryngeal artery (SLA). The SLA runs beneath the OOM, progressing to the medial side, where SLA penetrates the pars peripheralis of the OOM and travels between the pars paripheralis and pars marginalis. In proximity to the midline, the SLA runs more superficially along the dry mucosa. Consequently, injections at Points 4, 5, 8,and 9 are deemed very safe from vascular side effects. The subsequent safer areas include Points 1, 2, 7, and 8, while the most precarious part is considered to be Point 3. *, Mainly located arteries.

Sonographic results revealed that the labial arteries originated from the deep layers and exhibited a gradual transition towards the superficial layer, moving towards the midline (Figure 5).

### Sonography From the Oral Commissure to Point 1 (0-I) of the Upper Lip

**Figure 6.**
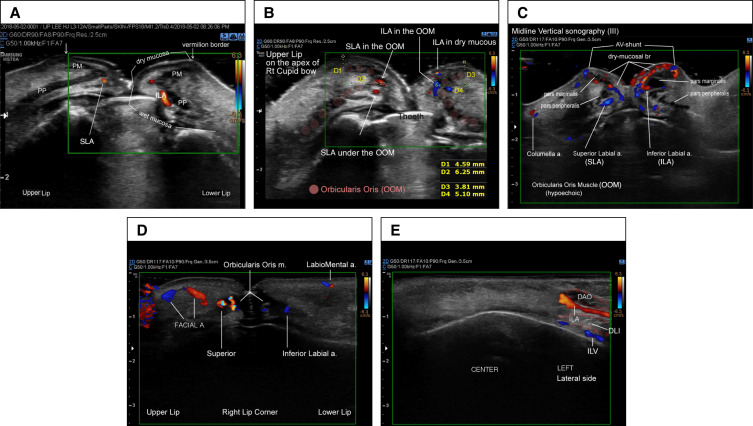
Sonographic findings. (A) Sonography at entry points 1 and 2. (B) Sonography on the vertical view of the right apex of Cupid's bow. (C) Identification of arteriovenous shunts of the labial arteries on the midline in sonography. (D) The facial artery, ILA, and SLA are located around the corners of the mouth. (E) Pathway of the inferior labial artery. DAO, depressor anguli oris; DLI, depressor labii inferioris; ILA, inferior labial artery; OOM, orbicularis oris muscle; PM, pars marginalis musculi orbicularis oris; PP, inferior labial artery; SLA, superior labial artery.

Subsequently, SLAs were found to be located in the wet mucosa (under the orbicularis oris muscle [OOM]) in 84% of cases, inside or between the OOM in 18% of cases, and in the dry mucosa (above the OOM) in only 6% of cases. Consequently, injection points 4 and 5 were identified as a very safe zone, minimizing the risk of bruising, because the SLA primarily courses beneath the OOM, and the injection site is positioned at a significant distance from the SLA (Figure 6A; Videos 1, 2).

### Sonography at Entry Point 1 (I)

At entry point 1, the SLAs were observed beneath the OOM near the wet mucosa in 78% of cases, inside the OOM in 44% of cases, and above the OOM (near the dry mucosa) in 8% of cases in this study. Furthermore, SLAs were observed to run both under and inside the OOM in 8% of cases. Consequently, when employing the 9-PIT, points 1 and 2 were determined to be at a safe distance from the SLA. The technique is considered secure because the injection layer is the submucosa, and the SLAs predominantly ran beneath the OOM in the majority of cases. Additionally, the pinch technique can help mitigate vascular side effects through compression and lifting. Compression collapses the vessel while lifting ensures that the injection area (the needle) is positioned away from the vessels. Mucosal branches and perforating arteries above the OOM were rarely found at points 1 and 2 (Figure 6A).

### Sonography at Apex of Cupid's Bow (II)

In vertical sonographic view at the right apex of Cupid's bow (the ending area of injection from points 1 and 2), SLAs were observed to run under the OOM (wet mucosa) in 48%, inside the OOM in 82% of cases, and over the OOM (within the dry mucosa) in 28% of cases in this study. The SLAs showed a more superficial course on the medial side than on the lateral side (Figure 6B).

### Sonography at Midline of Upper Lip (III)

In sonography, mucosal branches along dry mucosa and small perforating arteries in the midline of the upper lip were found in the dry mucosa in 80% of cases. This marks the first documentation of such sonographic findings in actual patients who underwent lip augmentation. Notably, point 3 emerged as the most vulnerable part for bruising in the 9-PIT, given the demonstration of an arteriovenous shunt in the midline of the upper lip via microcomputed tomography (micro-CT).^12^ The presence of an arteriovenous shunt in the midline of the upper and lower lips was observed through sonography for the first time in this study. Whether it is a superficial mucosal branch or an arteriovenous shunt, these arteries in the midline should be avoided during injection. The pinch technique proves valuable in reducing the risk of vascular injection. Additionally, the SLA was found to run more superficially on the medial side of lips than on the lateral side in sonography (Figure 6C; Video 3).

### Sonography at the Oral Commissure of the Upper Lip (0)

Injection near the corners of the upper lips is deliberately avoided. At points 4 and 5, SLAs were consistently observed under the OOM in all cases (100%) through sonography. Facial arteries are positioned laterally to the commissure, running cephalically and obliquely. Importantly, the facial arteries also are distant from the injection sites of the lip (Figure 6D; Video 2).

### Sonography at Points 8 and 9 (0-I): Lower Lip

The facial artery issued a branch identified as the inferior labial artery (ILA) situated behind (lateral to) the depressor anguli oris (DAO), extending anterosuperioly. Subsequently, the ILA proceeded medially (slightly upward and forward), coursing beneath the DAO and above the depressor labii inferioris. Following this path, the ILA intricately pierced the pars peripheralis of the OOM in a tortuous course. Continuing its course, the ILA ran beneath the pars marginalis of the OOM along the vermilion border of the lower lip, between the pars marginalis of the OOM. Consequently, injections at points 8 and 9 are deemed very safe in terms of vascular side effects. Although very small perforator arteries supplying the mucosal membrane were observed in sonography, the ILA appeared to consistently run beneath the OOA, with only a 4% exception (Figure 6E; Video 2).

### Sonography at Points 6 and 7 (II)

The ILA was observed to run under the OOM in the wet mucosa in 52% of cases, inside or between the OOM (between the pars marginalis and the pars peripheralis) in 84% of cases, and above the OOM (in the dry mucosa) in 24% of cases in this study (Figure 6B; Video 4).

### Midline of the Lower Lip (III)

In 43 cases (86%), the superficial dry mucosal branch was identified in the midline of the lower lips. Similar to the upper lips, small arteries in the lower lips exhibited anastomosis with the veins. Applying midline compression with a sterilized toothpick proved effective in preventing injections into the arteries. This method is extremely useful for reducing or preventing bruising, embolism, and skin necrosis while injecting in the lower tubercles of the lower lips. While the presence of an arteriovenous shunt in the midline of the upper lip was found in micro-CT,^12^ this study marks the first identification of an arteriovenous shunt in the midline of the lower lip through sonography. Whether it is the superficial mucosal branch or an arteriovenous shunt, caution is advised to avoid injection into these arteries in the midline (Figure 6C; Video 3).

### Sonography at the Oral Commissure of the Lower lip (0)

**Figure 7.**
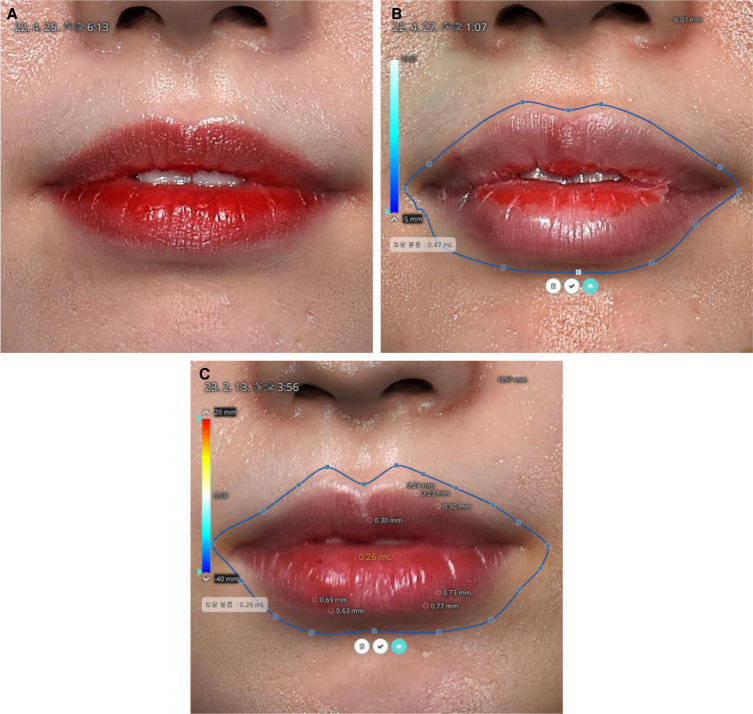
The shape and volume of the lip 2 days and 10 months after 0.5 mL hyaluronic acid filler in a 24-year-old female (the same subject as shown in Figure 4). (A) Before injection. (B) The injected volume was 0.5 mL. The remaining volume measured by the 3D scanner was 0.47 mL 2 days after the treatment. The immediate measured volume was larger than 0.5 mL, and after 2 days the swelling had subsided. (C) The injected volume was 0.5 mL. The remaining volume measured by the 3D scanner was 0.26 mL 10 months after the treatment. Half the originally injected volume was absorbed, and half persisted. The ridges of the upper vermilion border and central tubercle were elevated by 0.3 mm compared to before the procedure. The lower tubercles were elevated by about 0.7 mm compared to before the procedure. The curved length of the upper peristomal borderline increased and was maintained even after 10 months.

In the oral commissure, the ILA was consistently found deep to the OOM in 100% of cases in this study (*P* < .001). The ILA originated caudally away from the lower lips, ensuring the 9-PIT does not cause arterial injury near the oral commissure (Video 2; Figure 7).

### Sonography of the SLA and ILA

Both the SLA and ILA were observed to run under the OOM near the lip corners. They continued their course within the OOM and then traversed more superficially in the dry mucosal layer in the midline. Consequently, with the exception of the midline, the 9-PIT is considered safe from arterial injury (Figure 4; Video 4).

### Angle of Lip Corner

The angle of the lip corner decreased by 8.80% [3.71%] (mean [standard deviation]), shifting from 158.7° to 144.4° after 1 week (*P* < .001) (Figure 3).

### Extent of Lift of the Oral Commissure Using a Computer Program

The 3D scanner analysis revealed that the oral commissure was lifted by 1.02 [0.31] mm after 1 week (Figure 4).

### Length and Shape Along the Peristomal Border on the Upper Lips

The shape of the upper peristomal border became more exaggerated after the 9-PIT, with the length of the upper peristomal border (from central tubercle to oral commissure) increasing by 6.50% [4.65%] after 1 week (*P* < .001).

The curved S-shape of the upper lip line became more pronounced, indicating a lift of the lip corners (Figures 4, 7).

### GAIS

The GAIS score was 2.8 at 1 week postinjection, with 45 patients (90%) expressing extreme satisfaction with the results. The GAIS score decreased to 2.4 at 1 month and further to 1.9 at 3 months.

In 5 patients (10%), the areas injected at points 4 and 5 appeared as blisters when they returned after 1 week. Pressure applied with a cotton swab alleviated the blisters in 4 patients (8%), and in 1 case (2%), the blister nodules were successfully removed through simple squeezing with a 23G needle.

Eight patients (16%) expressed concerns about the initial results appearing too voluminous (overdose) the day after the procedure and expressed a desire to remove the HA filler with hyaluronidase. They were informed that the initial swelling resulting from the procedures could make the lips appear larger than the final expected results. Consequently, they were advised to wait without intervention. After 1 week, all patients reported that the swelling had subsided, and they were satisfied with the final results at the 1-week posttreatment visit.

No serious complications, including infection, prolonged swelling, Tyndall effect, or asymmetry, were observed. The initial injected amount (1 mL) had decreased by half (0.5 mL) after 1 year in most cases. Specifically, the volume of the initially injected 0.5 mL was further reduced to 0.26 mL after 10 to 12 months. Long-term changes in volume will be explored in detail in a future study.

## DISCUSSION

The 9-PIT encompasses Ar-LTIs (at points 1, 2, 3, 6, 7), Re-LTIs (at points 8, 9), retrograde fanning (at points 4, 5), and anterograde fanning (at point 6, 7). Linear threading at points 1, 2, 8, 9 involves the full insertion of the needle length (1 inch). Additionally, at points 3, 4, 5, 6, 7 the procedure is performed to enhance or create tubercles by inserting the needle from the entry point to the wet-dry junction.

Anterograde injection involves the extrusion of the filler while advancing the needle submucosally in the lip. Ar-LTI proves beneficial in enhancing features such as the vermilion border when moving toward Cupid's bow from points 1, 2. The presence of HA materials in front of the sharp needle tip facilitates pushing fine vessels out of their pathway. Ar-LTI can minimize the risk of vascular injury by preventing direct contact of the needle tip with the vessels through the HA material, thereby reducing the risk of intravascular injection. During Re-LTI, the injection of the filler material and the resulting changes (inflation) in the lips can be observed more easily while withdrawing the needle from the tissue. Re-LTI is particularly useful for softer tissue areas at points 4, 5, 8, 9.

Elevating the corners of the mouth using only fillers can be challenging. To achieve the appearance of higher mouth corners, the author experimented with creating a new upper lateral tubercle and lowering the side of the oral commissure (points 4 and 5). While points 8 and 9 naturally push the oral commissure upward, causing a subtle rise, the primary focus was on utilizing fillers alone. Although botulinum toxin injections weakening the DAO can enhance the effect achieved with the 9-PIT, this study exclusively demonstrates outcomes achieved with fillers.

Adequate needle depth can be discerned through visual cues. If the needle becomes visible through the mucosa, it indicates that the location is too superficial for the placement, posing a higher risk of creating a blister or Tyndall effect.^13^ Resistance to needle advancement may be observed if the needle is moved through a too-superficial layer. Additionally, an excessively superficial injection in the lips may cause mucosal blanching. If blanching occurs, the injection should be halted, and the HA filler material should be molded into the subdermal layer by applying gentle pressure with a cotton ball for 1 minute.^14^ Molding is not a necessity if the injection is executed correctly. It is employed selectively in cases of asymmetry or when the filler needs adjustment. While achieving perfection in every injection is not always possible, the goal is to perform injections proficiently, minimizing the need for subsequent molding. Conversely, if the injection is placed too deep into the muscle layer, detecting changes in soft tissue inflation becomes challenging. Injecting into the OOM makes shaping the lips even more difficult. Additionally, injecting filler into the OOM may result in the material shifting with the strong contraction of the muscle.

## CONCLUSIONS

By meticulously observing the blood vessel pathways in all patients by sonography, before and after filler injections, the author concludes that the 9-PIT is a safe method, effectively reducing the risk of vascular injuries. The author introduced a novel method to create lateral tubercles, giving the illusion of lifted lip corners. The 9-PIT demonstrated easy reproducibility for lifting lip corners and creating balanced and aesthetically pleasing lip augmentation. One notable advantage of this technique is the consistent achievement of expected results. This study introduces an innovative approach to lip filler injections, incorporating the concept of “lip occlusion,” which involves injecting only the upper lip while avoiding the lower lip region.

## Supplemental Material

This article contains supplemental material located online at www.aestheticsurgeryjournal.com.

## Supplementary Material

sjae086_Supplementary_Data

## References

[1] Kim J. Effects of injection depth and volume of stabilized hyaluronic acid in human dermis on skin texture, hydration, and thickness. Arch Aesthetic Plast Surg. 2014;20(2):97–103. doi: 10.14730/aaps.2014.20.2.97

[2] Kim J. Clinical effects on skin texture and hydration of the face using microbotox and microhyaluronicacid. Plast Reconstr Surg Glob Open. 2018;6(11):e1935. doi: 10.1097/GOX.0000000000001935 30881778 PMC6414123

[3] Kim J. Fine wrinkle treatment and hydration on the facial dermis using HydroToxin mixture of MicroBotox and MicroHyaluronic acid. Aesthet Surg J. 2021;41(6):NP538–NP549. doi: 10.1093/asj/sjaa231 32779694 PMC8240748

[4] Tran C, Carraux P, Micheels P, Kaya G, Salomon D. In vivo bio-integration of three hyaluronic acid fillers in human skin: a histological study. Dermatology. 2014;228(1):47–54. doi: 10.1159/000354384 24503674

[5] Wang F, Garza LA, Kang S, et al In vivo stimulation of de novo colla gen production caused by cross-linked hyaluronic acid dermal filler injections in photodamaged human skin. Arch Dermatol. 2007;143(2):155–163. doi: 10.1001/archderm.143.2.155 17309996

[6] Beer K, Glogau RG, Dover JS, et al A randomized, evaluator blinded, controlled study of effectiveness and safety of small particle hyaluronic acid plus lidocaine for lip augmentation and perioral rhytides. Dermatol Surg. 2015;41(Suppl 1):S127–S136. doi: 10.1097/DSS.0000000000000199 25828037

[7] Choi Y-J, We Y-J, Lee H-J, et al Three-dimensional evaluation of the depressor anguli oris and depressor labii inferioris for botulinum toxin injections. Aesthet Surg J. 2021;41(6):NP456–NP461. doi: 10.1093/asj/sjaa083 32232427

[8] Moradi A, Shirazi A. A retrospective and anatomical study describing the injection of botulinum neurotoxins in the depressor anguli oris. Plast Reconstr Surg. 2022;149(4):850–857. doi: 10.1097/PRS.0000000000008967 35139057

[9] Penna V, Stark GB, Voigt M, Mehlhorn A, Iblher N. Classification of the aging lips: a foundation for an integrated approach to perioral rejuvenation. Aesthetic Plast Surg. 2015;39(1):1–7. doi: 10.1097/PRS.0000000000005589 25409624

[10] Hegyi AN, Yablonovitch E. Nano-diamond imaging: a new molecular imaging approach. Proceedings of the Annual International Conference of the IEEE Engineering in Medicine and Biology Society (EMBC ‘12); September 2012; San Diego, Calif, USA. pp. 2639-2642. doi: 10.1109/EMBC.2012.6346506 23366467

[11] Online protractor. Accessed December 14, 2023. https://www.ginifab.com/feeds/angle_measurement/online_protractor.ko.php

[12] Yamamoto M, Chen H-K, Hidetomo H, et al Superior labial artery and vein anastomosis configuration to be considered in lip augmentation. Ann Anat. 2022;239:151808. doi: 10.1016/j.aanat.2021.151808 34324994

[13] Hirsch RJ, Narurkar V, Carruthers J. Management of injected hyaluronic acid induced Tyndall effects. Lasers Surg Med. 2006;38(3):202–204. doi: 10.1002/lsm.20283 16485276

[14] Rohrich RJ, Ghavami A, Crosby MA. The role of hyaluronic acid fillers (restylane) in facial cosmetic surgery: review and technical considerations. Plast Reconstr Surg. 2007;120(6 Suppl):415–454S. doi: 10.1097/01.prs.0000248794.63898.0f 18090342

