# Supplementary figures and images for: 9-point Injection Technique for Lip Augmentation and Lip Corner Lifting Using Sonographic Imaging of the Labial Artery Pathway
Source: Aesthet Surg J. 2024 Apr 23;44(10):1080–90. doi: 10.1093/asj/sjae086 (PMC11483566; doi:10.1093/asj/sjae086)

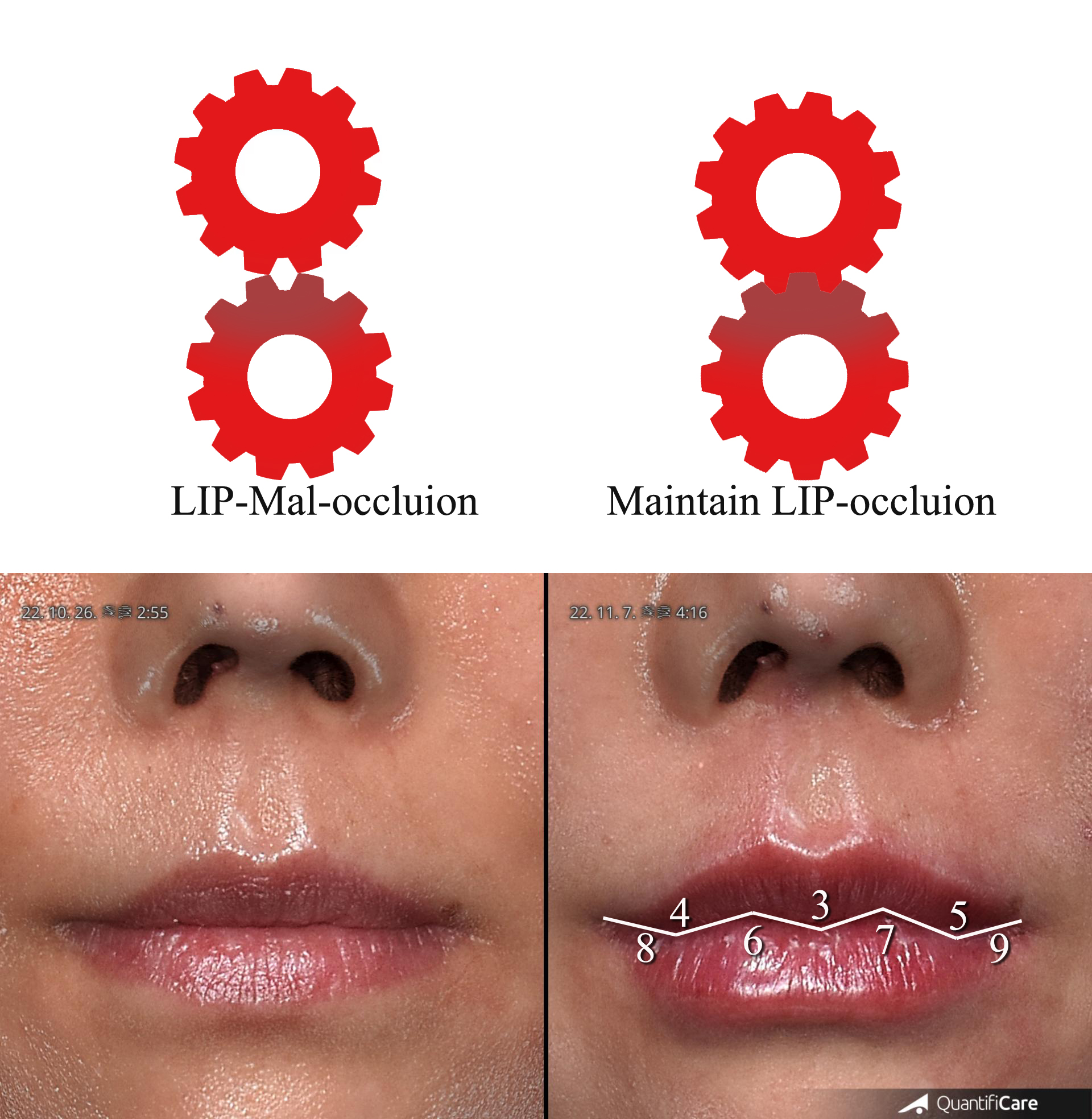

Supplement: sjae086_Supplementary_Data [file sjae086_Supplementary_Data.zip › Supplemental Figure 1.jpg]
